# Magnetic resonance imaging-guided stereotactic body radiotherapy for prostate cancer (mirage): a phase iii randomized trial

**DOI:** 10.1186/s12885-021-08281-x

**Published:** 2021-05-11

**Authors:** Ting Martin Ma, James M. Lamb, Maria Casado, Xiaoyan Wang, T. Vincent Basehart, Yingli Yang, Daniel Low, Ke Sheng, Nzhde Agazaryan, Nicholas G. Nickols, Minsong Cao, Michael L. Steinberg, Amar U. Kishan

**Affiliations:** 1 Department of Radiation Oncology, University of California Los Angeles, 200 Medical Plaza Driveway, Suite # B265, Medical Plaza Driveway, Los Angeles, CA 90095 USA; 2 Department of Medicine Statistics Core, University of California Los Angeles, 200 Medical Plaza Driveway, Suite # B265, Medical Plaza Driveway, Los Angeles, CA 90095 USA; 3 Department of Urology, University of California Los Angeles, 200 Medical Plaza Driveway, Suite # B265, Medical Plaza Driveway, Los Angeles, CA 90095 USA

**Keywords:** Prostate Cancer, Stereotactic body radiotherapy (SBRT), Magnetic resonance imaging (MRI), Computed tomography (CT), Genitourinary (GU) toxicity, Gastrointestinal (GI) toxicity

## Abstract

**Background:**

Stereotactic body radiotherapy (SBRT) is becoming increasingly used in treating localized prostate cancer (PCa), with evidence showing similar toxicity and efficacy profiles when compared with longer courses of definitive radiation. Magnetic resonance imaging (MRI)-guided radiotherapy has multiple potential advantages over standard computed tomography (CT)-guided radiotherapy, including enhanced prostate visualization (abrogating the need for fiducials and MRI fusion), enhanced identification of the urethra, the ability to track the prostate in real-time, and the capacity to perform online adaptive planning. However, it is unknown whether these potential advantages translate into improved outcomes. This phase III randomized superiority trial is designed to prospectively evaluate whether toxicity is lower after MRI-guided versus CT-guided SBRT.

**Methods:**

Three hundred men with localized PCa will be randomized in a 1:1 ratio to SBRT using CT or MRI guidance. Randomization will be stratified by baseline International Prostate Symptom Score (IPSS) (≤15 or > 15) and prostate gland volume (≤50 cc or > 50 cc). Five fractions of 8 Gy will be delivered to the prostate over the course of fourteen days, with or without hormonal therapy and elective nodal radiotherapy (to a dose of 5 Gy per fraction) as per the investigator’s discretion. The primary endpoint is the incidence of physician-reported acute grade ≥ 2 genitourinary (GU) toxicity (during the first 90 days after SBRT), as assessed by the CTCAE version 4.03 scale. Secondary clinical endpoints include incidence of acute grade ≥ 2 gastrointestinal (GI) toxicity, 5-year cumulative incidences of physician-reported late grade ≥ 2 GU and GI toxicity, temporal changes in patient-reported quality of life (QOL) outcomes, 5-year biochemical recurrence-free survival and the proportion of fractions of MRI-guided SBRT in which online adaptive radiotherapy is used.

**Discussion:**

The MIRAGE trial is the first randomized trial comparing MRI-guided with standard CT-guided SBRT for localized PCa. The primary hypothesis is that MRI-guided SBRT will lead to an improvement in the cumulative incidence of acute grade ≥ 2 GU toxicity when compared to CT-guided SBRT. The pragmatic superiority design focused on an acute toxicity endpoint will allow an early comparison of the two technologies.

**Trial registration:**

Clinicaltrials.gov identifier: NCT04384770. Date of registration: May 12, 2020.

https://clinicaltrials.gov/ct2/show/NCT04384770

**Protocol version:**

Version 2.1, Aug 28, 2020.

**Supplementary Information:**

The online version contains supplementary material available at 10.1186/s12885-021-08281-x.

## Background

With a combination of high incidence rate, high rate of cure, and treatment-associated morbidity, prostate cancer is ranked as the leading cause of cancer treatment–related years lived with disability (YLD) worldwide [1]. In the United States, nearly 192,000 men will be diagnosed with prostate cancer in 2020, most of whom will be diagnosed with clinically localized disease [2]. Besides active surveillance for highly selected groups of patients (very low-risk/low-risk and certain favorable intermediate-risk), commonly used definitive treatment modalities include radical prostatectomy, external beam radiotherapy (EBRT) and brachytherapy [3]. Various EBRT regimens are now supported by entities such as the National Comprehensive Cancer Network (NCCN), ranging from conventionally-fractionated radiotherapy (consisting of small daily doses per day over the span of 39–45 treatments) to stereotactic body radiotherapy (SBRT, consisting of large daily fractions given in as few as four-to-five treatments) [3–6].

Given the high cure rate and long life-expectancy following treatment, quality of life (QOL) often remains the paramount factor in decision making [7–12]. After definitive EBRT, a major concern is late genitourinary (GU) toxicity [13]. Physician-scored toxicity measures using standardized scoring systems such as Common Terminology Criteria for Adverse Events (CTCAE) are important for standardizing adverse event reporting after therapy and can create an objective rubric for comparing treatment outcomes. Acute GU toxicity, defined as adverse events occurring within the first 90 days after completion of radiation, is typically characterized by “urinary bother” symptoms, including frequency, urgency, reduced flow, and dysuria. These toxicities occur in nearly 30% of patients receiving modern radiotherapy [4] and resolve within several months. Late GU toxicities can include dysuria, frequency, urgency, contracture, spasm, reduced flow, hematuria, and rarely, fistulization, necrosis, ulceration, and incontinence. The 5-year late grade ≥ 2 GU toxicity rates range from 12 to 15% [14–18]. However, the cumulative incidence insidiously increases over time with 10-year rates of 17–20% reported on clinical trials [6, 19]. Registry data have also identified that the risk of significant toxicity in patients receiving prostate EBRT, when compared to patients who have not received this, remains elevated for at least 10 years [20].

As SBRT rises to the forefront as a preferred radiation modality for localized PCa, it is critical to rigorously evaluate its toxicity profile, with the hopes of achieving better treatment. The importance of technology can be underscored by reviewing toxicity rates over time as different technological advancements have been deployed. The rates reported in the recently reported PACE-B trial [4] and in the pooled SBRT consortium [5] are much lower than those reported in the seminal HYPO-RT-PC trial [6]. The rates of acute and late/cumulative toxicities in these three studies were summarized in Table 1. For example, there were lower rates of acute grade ≥ 2 Radiation Therapy Oncology Group (RTOG) GU (20.3% vs. 28%) and GI (9.0% vs. 24%) toxicities in PACE-B than the HYPO-RT-PC trial. The pooled SBRT consortium showed the lowest acute grade ≥ 2 GU and GI toxicities (9.6 and 3.4%, respectively). It also reported a much reduced 7-year cumulative incidence of late grade ≥ 3 GU (2.4% vs. 4.2%) and GI (0.4% vs. 1.7%) toxicities than the HYPO-RT-PC’s 5-year cumulative incidence. An important factor to consider is that the radiation planning techniques used in the landmark HYPO-RT-PC trial are outdated, including three-dimensional conformal radiotherapy and the use of large 7 mm isotropic margins. In contrast, the PACE-B trial and the trials included in the SBRT consortium employed modern SBRT techniques including intensity-modulated radiotherapy (IMRT). The difference in the treatment toxicities is potentially a reflection of the use of IMRT. However, only 73% of patients having received SBRT had implanted fiducial markers, and a minority (41.7%) had motion monitoring during treatment. The treatment patients received in the pooled SBRT consortium were closest to modern SBRT delivery. For instance, all patients had implanted fiducial markers to guide motion management and 88% of men had intrafraction prostate motion monitoring (with KV imaging or transponder beacon) during radiation. The majority of the remainder had at least interval imaging to account for prostate motion during treatment, and margins ranged from 2 mm to 5 mm isotropically. Indeed, the absolute grade ≥ 3 toxicity rates seen at seven years was over three-fold less than in the HYPO-RT-PC trial, which may be attributable to these technological improvements.

**Table 1:** Important studies regarding the acute and late/cumulative toxicities for prostate SBRT

Study	Number of pts	Risk group	Arms	Acute toxicities	Late/cumulative toxicities
HYPO-RT-PC (2005–2015)	1200	Intermediate-risk: 89% high-risk: 11%	UF-RT: 42.7 Gy in 7 fx (6.1 Gy/fx), delivered over 2.5 weeks vs. CF-RT: 79 Gy in 38 fx (2 Gy/fx)	Acute RTOG grade ≥ 2 GU toxicity: 28% vs. 23%; acute RTOG grade ≥ 2 GI toxicity: 24% vs. 24%.	5-year grade ≥ 2 GU toxicity: 18% vs. 17%; 5-year grade ≥ 2 GI toxicity: 10% vs. 10%; 5-year grade ≥ 3 GU toxicity: 4.2% vs. 4.7%; 5-year grade ≥ 3 GI toxicity: 1.7% vs. 1.9%
PACE-B (2012–2018)	874	Low risk: 8%; intermediate-risk: 92%	UF-RT: 36.25 Gy in 5 fx (7.25 Gy /fx delivered consecutively [20.7%] or over the span of ~ 2 weeks [79.3%]) vs. CF-RT: 78 Gy in 39 fx (2 Gy/ fx)-31% or MF-RT: 62 Gy in 20 fx (3.1 Gy/fx)- 69%	Acute RTOG grade ≥ 2 GU toxicity exceeding baseline: 20.3% vs. 24.9%; Acute RTOG grade ≥ 2 GI toxicity: 9.0% vs. 12.2%	N/A
Pooled SBRT consortium (12 single arm phase II studies between 2000 and 2012)	2142	low-risk: 55.3%; favorable intermediate-risk: 32.3%; unfavorable intermediate-risk: 12.4%	UF-RT: 38 Gy in 4 fx (9.5 Gy/ fx) or 40 Gy in 5 fx (8 Gy/fx)	Acute grade ≥ 2 GU toxicity*: 9.6%; acute grade ≥ 2 GI toxicity*: 3.4%. Acute grade ≥ 3 GU toxicity*: 0.6%; acute grade ≥ 3 GI toxicity*: 0.09%.	7-year cumulative incidence of late grade ≥ 3 GU toxicity: 2.4%; grade ≥ 3 GI toxicity: 0.4%.

*Toxic event scoring derived per institutional or clinical trial protocols, which is a combination of CTCAE and RTOG defined events

Despite these technological improvements, the incidences of acute and late grade ≥ 2 toxicities are not inconsequential. The best estimates of grade ≥ 2 toxicities following modern SBRT come from the PACE-B trial (acute) [4] and a multi-center SBRT trial run in the United States [14]. Overall, one would expect modern SBRT to have acute grade ≥ 2 GU and GI toxicity rates of 27.4 and 15.3%, and late grade ≥ 2 GU and GI toxicity rates of 13.3 and 2.0% at 5 years. The persistent rate of toxicity may be driven by a host of factors, including intrinsic radiosensitivity and the dose of radiation delivered to relevant adjacent organs-at-risk, including the bladder, urethra, and rectum. Indeed, because of prostatic motion— both intrafraction (during fractions) and interfraction motion (between fractions) — margins must be placed around the prostate when targeting it with external radiation to assure adequate tumor dosing. This leads to the irradiation of portions of adjacent organs, such as the bladder and rectum. It is known that doses, particularly high doses delivered to small areas, are primary drivers of post-radiation GU and GI toxicities [21]. In addition to uncertainties due to target motion, additional margins must be placed around the prostate target because of geometric uncertainty and patient setup errors.

MRI-guided linear accelerator (LINAC) systems, which have just recently become commercially available, offer several technical advantages that can minimize the planning margins needed for SBRT. First, these devices can monitor prostate motion in real-time, abrogating the need for implanted fiducials and eliminating the need for frequent X-ray based imaging to detect these fiducials. The precise visualization of boundaries between the prostate and nearby important healthy tissues such as the bladder and rectum from the MR images also allows for narrower margins. Second, the prostate is seen more clearly on MRI than on CT-based imaging, such that prostate target volumes generated by MRI are smaller and more reproducible than those generated by CT [22]. While MRIs can guide the generation of CT-based contours through image fusion, the fusion process itself introduces 1–2 mm of residual error [23]. Treatment on an MRI-based device, which itself has significantly improved soft-tissue contrast, allows for more accurate target delineation by bypassing the need for CT-MRI fusion. Finally, the adjacent critical structures are highly deformable and, despite rigorous patient preparation instructions, will lead to anatomic variability from fraction to fraction. Such deformation cannot be corrected using fiducial marker-based translational and rotational corrections on a conventional LINAC with x-ray image guidance. Daily cone-beam CT (CBCT) is not well equipped for this task either due to poor organ visualization. Modern MRI-guided LINAC systems can uniquely correct for these deformations using online adaptive radiotherapy, wherein a new radiotherapy plan is generated on the basis of the anatomy seen at a given fraction [24]. PTV margin, even by 1–2 mm, has a dramatic impact on PTV volume. For instance, for a spherical target with radius ~ 2.28 cm, the volume is 50 cc. A 4 mm PTV margin would lead to a volume of 81 cc, compared with 64 cc for a 2 mm PTV. Considering the majority of the excess PTV will be affecting the bladder wall and rectum, this difference is considerable.

Despite these theoretical advantages, there are limited data to support a meaningful clinical improvement with MRI-guided SBRT versus standard CT-guided SBRT. A recently reported phase II trial (NCT03961321) of 101 patients [25] by Bruynzeel et al. represents the only prospective data to date on MRI-guided SBRT. The authors delivered SBRT (36.25Gy in 5 fractions) under MRI guidance to the target volume with daily plan adaptation, while attention was paid to limit the total dose to the urethra to 32.5Gy. Acute CTCAE version 4.0 grade ≥ 2 GU toxicity incidence was 19.8% at the end of SBRT, while acute CTCAE version 4.0 grade ≥ 2 GI toxicity was 3.0%. These compare favorably to the corresponding rates of 27.4 and 15.3% in PACE-B [4]. Given these provocative results, it is imperative to verify whether MRI-guided SBRT truly offers an improved toxicity profile over CT-guided SBRT. The purpose of this randomized trial is to rigorously answer this question.

## Methods/design

**Fig. 1:**
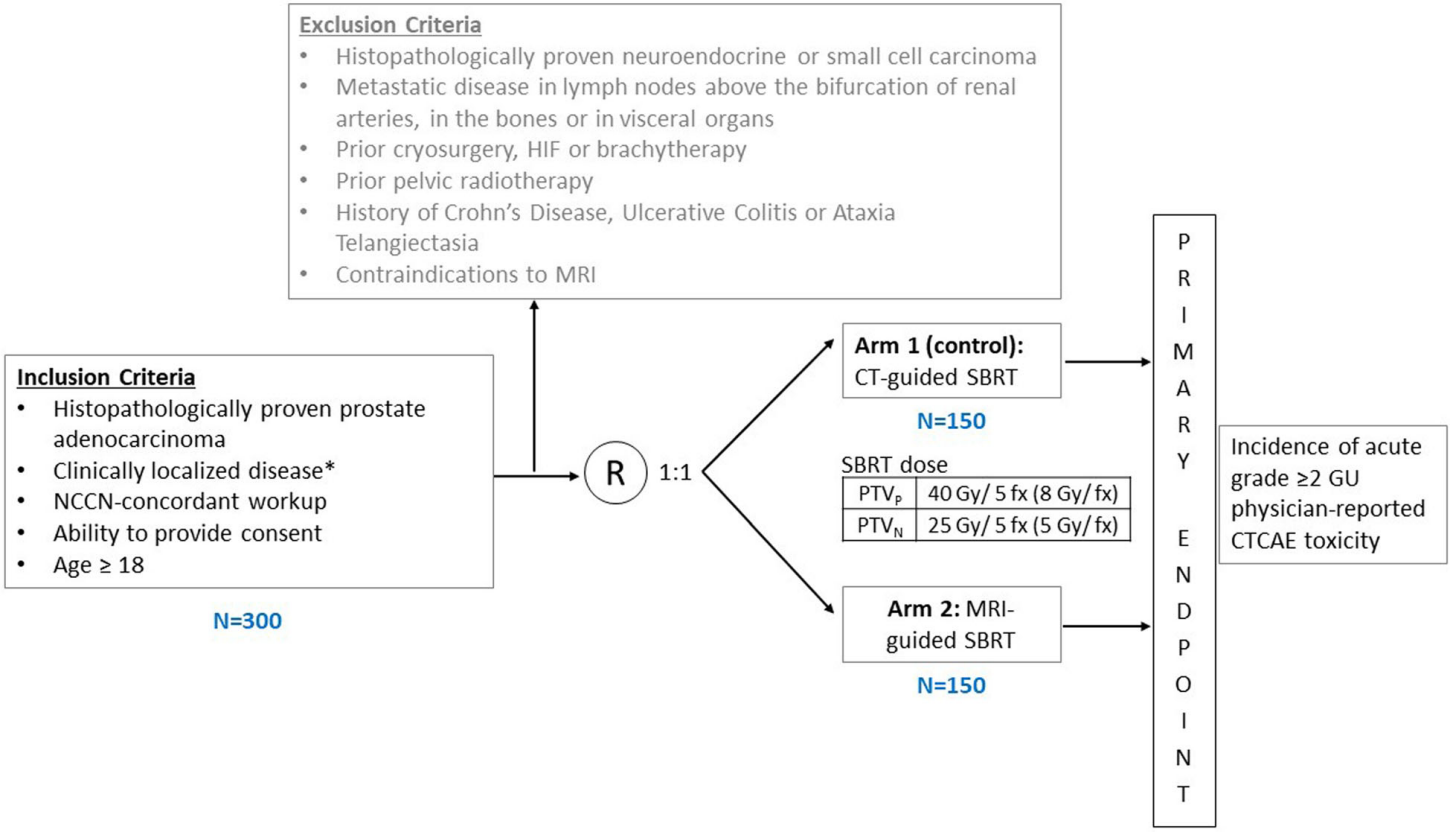
Trial Schema. * Nodal disease (N1/M1a) identified on a Prostate-specific membrane antigen (PSMA) PET/CT scan

This is a non-blinded single-center randomized trial designed to evaluate the superiority of MRI-guided SBRT over standard CT-guided SBRT for PCa in terms of acute physician-scored GU toxicity. Acute physician-scored GI toxicity, late physician-scored toxicity and patient-reported QOL, and five-year biochemical recurrence-free survival (BCRFS) will also be evaluated. We plan to enroll 300 patients with an expected rate of accrual in the range of 100 patients per year. Standard of care SBRT for localized PCa will be delivered either via an MRI-guided LINAC or a CT-guided LINAC. Five fractions of SBRT will be delivered within the span of 14 days (no more frequent than every other day), and patients will be followed for 5 years per routine standard of care. The trial schema is displayed in Fig. 1.

### Ethics approval

The study is approved by the Institutional Review Board (IRB) of the University of California Los Angeles (UCLA IRB #20–000328). The MIRAGE trial is registered at the US National Institutes of Health (ClinicalTrials.gov) #NCT04384770. The current protocol is version 2.1 dated August 28, 2020.

### Objectives

The primary objective of this study will be to determine whether MRI-guided SBRT can lead to a 14% absolute reduction in the cumulative incidence of acute physician-scored ≥2 GU toxicity (defined by the CTCAE version 4.03) when compared to a rate of 29% in CT-guided SBRT group, corresponding to a relative risk reduction of 48%.

#### Primary endpoint

The incidence of acute grade ≥ 2 GU physician-reported toxicity, as assessed by the CTCAE version 4.03 scale. The timeframe will be restricted to the first 90 days after SBRT.

#### Secondary endpoints

1. The incidence of acute grade ≥ 2 GI toxicity as assessed by the CTCAE version 4.03 scale. The timeframe will be restricted to the first 90 days after SBRT.
2. The 5-year cumulative incidences of late grade ≥ 2 GU and GI physician-reported toxicity, as assessed by the CTCAE version 4.03 scale.
3. The temporal changes in patient-reported QOL outcomes will be obtained depending on the instrument used. For the Expanded Prostate Cancer Index-26 (EPIC-26) instrument, these will be represented by changes from baseline in the urinary incontinence, urinary obstruction, bowel, sexual function, and hormone/vitality domains. Changes will be analyzed with respect to whether they represent minimally important differences [26]. For the IPSS and Sexual Health Inventory for Men (SHIM) instruments, the numerical change from baseline, as well as the raw score at any given timepoint, will be extracted.
4. Five-year BCRFS, with biochemical recurrence (BCR) defined as serum PSA levels that are 2 ng/mL higher than the nadir PSA achieved after SBRT.
5. The proportion of fractions of MRI-guided SBRT in which online adaptive radiotherapy was used.

### Inclusion criteria

- Histologically confirmed, clinical localized adenocarcinoma of the prostate
- No evidence of metastatic disease in lymph nodes above the bifurcation of the renal arteries, or in bones or visceral organs (nodal disease identified on a Prostate-specific membrane antigen [PSMA] PET/CT scan below the bifurcation of the renal arteries are amenable)
- Staging workup as recommended by the NCCN on the basis of risk grouping:
  - Low risk: No staging workup required
  - Favorable intermediate-risk: CT abdomen/pelvis if Memorial Sloan Kettering Cancer Center (MSKCC) nomogram predicts > 10% probability of lymph node involvement
  - Unfavorable intermediate-risk: technetium bone scan, CT abdomen/pelvis if MSKCC nomogram predicts > 10% probability of lymph node involvement
  - High-risk: technetium bone scan, CT abdomen/pelvis if MSKCC nomogram predicts > 10% probability of lymph node involvement
  - Advanced imaging studies (i.e. PSMA PET/CT and Axumin PET/CT scan) can supplant a bone scan if performed first.
- Age ≥ 18
- Ability to understand, and willingness to sign, the written informed consent

### Exclusion criteria

- Patients with neuroendocrine or small cell carcinoma of the prostate
- Patients with any evidence of distant metastases except that evidence of lymphadenopathy below the level of the renal arteries can be deemed locoregional per the discretion of the investigator.
- Prior whole gland cryosurgery, high-intensity focused ultrasound (HIFU) or brachytherapy of the prostate
- Prior pelvic radiotherapy
- History of Crohn’s Disease, ulcerative colitis, or ataxia telangiectasia
- Contraindications to MRI, including:
  - Electronic devices such as pacemakers, defibrillators, deep brain stimulators, cochlear implants;
  - Metallic foreign body in the eye or aneurysm clips in the brain;
  - Severe claustrophobia

### Selection, study enrolment and randomization procedures

Patients seen as new patients in consultation in the UCLA Radiation Oncology clinic who are being evaluated for potential definitive radiotherapy for prostate cancer options will be informed of this clinical study if eligible. An informed consent form will be given to the patient for review. Upon confirmation of eligibility and enrollment in the study, the following will be obtained: (1) Medical history, clinical examination and consultation with Radiation Oncology; (2) Signed informed consent. Whenever feasible and in the best interest of the subject, the clinical examination, consultation and informed consent discussion and consent form signing may occur via telemedicine.

After the patient eligibility form is filled out in the Research Electronic Data Capture (REDCap) system, patients were randomly assigned to the two arms by a reproducible computer code developed by the UCLA Department of Medicine Statistics Core (DOMStat). To ensure balance between treatment allocation, stratified permuted blocked randomization will be used. The block sizes used will be blinded to the radiation oncology research team when enrolling a patient. The allocation result will not be released until after the screening/baseline data are filled out in REDCap (no anticipation of the group assignment will be possible). To obtain adequate “allocation concealment”, a list of random allocations has been created for patients 1 through 300. This list will be stored in REDCap and will not be modified.

Randomization will be stratified by the baseline IPSS (≤15 or > 15) and prostate gland volume (≤50 cc or > 50 cc). Prostate gland volume will be determined with MRI, which is mandatory. For analysis of the secondary endpoints of acute and late physician-scored toxicity, as well as bowel-related patient-reported outcomes, the analysis will be further stratified by the use of hydrogel spacers (use of spacer vs. no spacer used). Within each stratum, participants will be assigned in 1:1 ratio within each randomization block to one of the treatment arms:

1. Arm 1 (*n* = 150): The patient will undergo CT-guided SBRT.
2. Arm 2 (*n* = 150): The patient will undergo MRI-guided SBRT.

All the data management will be performed in the online clinical trial database. This is an open-label study. Trial participants, care providers, outcome assessors, and data analysts will be aware of the assignment after enrollment is completed. The randomization number and assignment will be communicated by phone or email to the treating physician. Patients will be informed by phone or email of the randomization assignment.

### Interventions

#### Study procedure

Enrolled patients will be treated with CT-guided SBRT on a standard, gantry-mounted LINAC (Arm 1) or with MRI-guided SBRT (Arm 2) on an MRI-guided LINAC (MRIdian System™, ViewRay™, Cleveland, OH, USA).

#### Radiation simulation planning and dosage

Enrolled patients will undergo CT simulation and planning as per routine for patients. Patients will be provided a bladder filling protocol with instructions to void one hour prior to simulation (and prior to each treatment), and drink at least two eight-ounce glasses of water. Patients will also be asked to perform a Fleet enema the night before and morning of their simulation scan. At the time of simulation, a custom VacLoc bag, alpha cradle, or equivalent device will be used for patient immobilization and establishment of treatment geometry. A pelvic CT without contrast will be performed for radiotherapy simulation (i.e., treatment planning CT) with a slice thickness of 1.5 mm. For patients enrolled on the MRI-guided SBRT arm, an additional MRI will be obtained in the treatment position on the MRI-guided LINAC.

Implanted fiducial markers are routinely used to assist with motion management when treating prostate cancer patients with any form of external radiotherapy, including SBRT [27]. These will be considered required for patients treated with CT-guided SBRT except in cases where a medical contraindication is present, as is consistent with our internal SBRT protocol. For patients being treated with MRI-guided SBRT, since the prostate can be visualized with the real-time cine feature, implanted fiducial markers will not be required. The use of a hydrogel is not an exclusion criterion for the trial, but is not considered mandatory either.

#### Radiotherapy contouring and planning

The entire prostate will be contoured as the CTV_P_. The proximal 1 cm of seminal vesicles will also be included in the CTV_P._ for patients with intermediate and high-risk disease. For patients with cT3a disease (i.e. extracapsular disease), the CTV_P_ will be expanded to cover the radiographic extent of ECE. For patients with cT3b disease, the CTV_P_ will be expanded to cover the entire seminal vesicle. The CTV_P_ will be expanded by 2–5 mm isotropically (typically 4 mm isotropically for CT-guided arm, per recent data from our institution [28] and 2 mm isotropically for the MRI-guided arm). Note the posterior expansion can be smaller than the expansion in other directions, i.e. the posterior expansion can be anisotropic with respect to the other expansions) to form the PTV_P._ We adopted a smaller margin of 2 mm for the MRI-guided arm given its ability for real-time tracking and superior prostatic anatomy visualization (i.e., greater contour certainty), with little concerns of marginal misses related to excess motion or undercontouring. For selected patients with high-risk disease, whole pelvic radiotherapy (WPRT) can be considered. If WPRT is to be delivered, the obturator, presacral, internal iliac, and external iliac nodal stations will be considered as targets. The nodal CTV (CTV_N_) will be defined as per the RTOG consensus guidelines [29]. CTV_N_ will be expanded isotropically by 4 mm to form PTV_N_.

Prescription doses for PTV_P_ and PTV_N_ are shown in Fig. 1. For all PTVs, the prescription dose will be prescribed such that 95% of the PTV receives at least the prescription dose, unless doing so would lead to violation of the organ-at-risk (OAR) dose constraints listed in Additional file 1. In such cases, undercoverage of either the PTV_P_ or the PTV_N_ will be allowed per physician discretion.

#### OAR doses

OAR doses are listed in Additional file 1. Doses that exceed the constraints below will be considered deviations from the protocol and can be delivered if study investigators agree that the deviation is acceptable and unlikely to cause excessive morbidity.

#### Radiation treatment delivery

The same patient immobilization, bladder filling and bowel preparation protocol for simulation will be employed during treatment delivery. Before each fraction of radiation treatment delivery, a 1.5 mm isotropic voxel size or better high resolution breathing scan will be taken with the on-board MRI to delineate the geometry of the target and OAR. If deemed necessary, online adaptive planning will be performed, wherein the planning GTVs, CTVs, and OARs are deformably transferred to the online MR images via registration, and the treatment plan is reoptimized to meet or exceed the original planning goals. Gating-based treatment, with gating based on the imaged location of the prostate organ or on rectal distention, can be employed at the discretion of the treating physician. Treatments will be delivered every other day, or consecutive days if necessary, with all fractions to be delivered within a period not exceeding 14 consecutive chronologic days.

#### Hormonal therapy (HT)

HT will be delivered at the discretion of the treating physician. Per the NCCN guidelines [3], HT is recommended at a duration of 4–6 months for unfavorable intermediate-risk prostate cancer or 12–36 months for high-risk prostate cancer (with 12–36 months in the context of extremely dose-escalated radiotherapy, and 18–36 months for standard dose-escalated radiotherapy). HT generally consists of combined androgen blockade, which is comprised of (a) a luteinizing hormone-releasing hormone agonist (e.g. leuprolide) or a gonadotropin-releasing hormone antagonist (e.g. degarelix) and (b) an oral anti-androgen (e.g. bicalutamide). In combined androgen blockade, the anti-androgen is generally given for one to six months. Given the emerging roles of advanced anti-androgen agents, particularly in high-risk disease [30], enhanced HT agents may also be given per physician discretion.

### Follow-up and adherence to interventions

Patients will be evaluated at their initial encounter (baseline/pre-treatment), at 1 month post-SBRT, at 3 months post-SBRT, and then every 3 months for the first year after treatment, and then every 6 months for a minimum of 5 years after treatment (+/− 4 weeks). After 5 years have elapsed, patients will be evaluated on an annual basis (+/− 4 weeks). Visits after 3 months, or at any timepoint at which the investigator believes it is in the best interest of the subject, may be performed remotely. No ancillary or post-trial care procedures are specified per the protocol.

### Physician scored toxicity

All acute and late adverse events from protocol radiation therapy will be reported and scored for severity using the CTCAE version 4.0.3. The same toxicity scale will be used for both acute and late radiation adverse events.

### Patient-reported toxicity

- The EPIC-26 questionnaire will be used to assess changes in the urinary incontinence, urinary obstruction, bowel, sexual function, and hormone/vitality domains [31]. Changes in domain scores at each time point will be classified as minimally important differences or not as previously reported [26].
- The IPSS questionnaire will be used to assess lower urinary tract symptoms [32]. Scores less than or equal to 7 indicate mild symptoms, scores ranging from 8 to 19 indicate moderate symptoms, and scores ranging from 20 to 35 indicate severe symptoms. A single question is included to inquire about the quality of life, and the answers range from “delighted” to “terrible” or from 0 to 6. Both changes in total IPSS scores from baseline and the total IPSS score at any given time point will be reported.
- The International Index of Erectile Function-5 (IIEF-5) or SHIM questionnaire will be used to assess erectile function [33]. The maximum score of 25 indicates maximal erectile function, while scores of 17 or lower indicate erectile dysfunction [34]. Both changes in total SHIM scores from baseline and the total SHIM score at any given time point will be reported.

### Treatment efficacy

Oncologic assessment for patients in this study will be consistent with patients managed with definitive radiotherapy for prostate cancer. This follow-up consists of PSA drawn every 3 months for the first year, then every 6 months until 5 years have passed since SBRT, and then once per year subsequently. Data of routine imaging (bone scan, CT or MRI) as clinically indicated will be collected on any patient who presents with any symptoms or PSA progression consistent with cancer recurrence. Recurrences will be managed according to the standard of care after primary radiotherapy for prostate cancer. Post treatment follow-up schedule is shown in Table 2.

**Table 2:** Study calendar

Procedure	Pre Study	Pre-RT (up to 4 months prior)	Baseline Pre-SBRT Day 1	On Tx SBRT 5 fx	POST TREATMENT VISITS (+/− 4 weeks)*									
					1 month (+/− 4 wks)	3 month (+/− 4 wks)	6 month (+/− 4 wks)	9 month (+/− 4 wks)	12 month (+/− 4 wks)	18 month (+/− 4 wks)	24 month (+/− 4 wks)	Q 6 month × 4 yrs		EOS Or Early Term Visit
												30 M 36 M 42 M	48 M 54 M 60 M (+/− 4 wks)	
Informed Consent	x													
Demographics	x													
Medical History	x													
SOC Physical Exam	x													x
Toxicity assessments / Quality of Life Questionnaires		x**	x**		x	x	x	x	x	x	x	x		x
SOC Non-Contrast Pelvic CT scan^***^		x												
SOC MRI of the Prostate or Pelvis		x												
SOC PSA Draw****		x				x	x	x	x	x	x	x		x
Translational saliva collection◦			x											
Record Radiation Therapy - 5 fractions				x										

* Follow-up visits can be conducted over the telephone, with remote collection of QOL information

**Toxicity and QOL assessment required either at pre-RT OR pre-SBRT, but not at both time points

*** For patients with PSA < 1.0 ng/mL, the treatment planning CT can substitute for a diagnostic CT scan (in this case, the CT simulation should be within 1 month of radiotherapy initiation)

**** These SOC blood samples can be drawn remotely, in the event that the patient is following up outside the UCLA system. In these cases, the lab reports should be provided to the study investigators

◦ **Whenever feasible, but may be waived**

EOS, end of study; SOC, standard of care

### Data management and confidentiality

The radiation oncology research staff will be responsible for the database records of study patients. The data will be kept on the research coordinator’s computer, under password protection, with the patient information de-identified (study patients will be referred by their coded study number). A chart with all the relevant research patient information will be maintained for each patient by the research coordinator, and will be filed in a firewall protected computer. Only the research team (study coordinator, investigators, and project supporting staff) will have the password and key to the data from the study patients. Specimens will be stored under the patient’s coded study number. The patient’s name or other public identifiers will not be included in any information shared with other investigators. The master key that will identify specific study patients to their coded study number will be kept in a separate password protected file on the research coordinator’s computer. Only the study coordinator and the principal investigator will know the password to this file. Because SBRT is considered a standard of care option in the United States, no independent data monitoring committee is needed as there are no patient safety issues involved. The trial is subject to audit per the discretion of the IRB at the University of California, Los Angeles.

### Statistical analysis

#### Sample size

The PACE-B trial [4] and a multi-center SBRT trial run in the United States [14] provided the best estimate of acute grade ≥ 2 toxicities following modern SBRT. Extrapolating from these two trials, one would expect modern SBRT to have acute grade ≥ 2 GU and GI toxicity rates of 29.1 and 16.0%, and 5-year incidences of late grade ≥ 2 GU and GI toxicity of 13.3 and 2.0%. Based on a recently reported phase II study, MRI-guided SBRT may lead to acute grade ≥ 2 GU and GI toxicity rates of 19.8 and 3.0% [25]. Given that these results were based on an early MRI-guided SBRT experience and the PTV margin used in the MRI-guided arm in the current study is smaller (2 mm instead of 3 mm), it is reasonable to anticipate an acute grade ≥ 2 GU toxicity rate of closer to 15%.

The primary endpoint for this study is the incidence of acute grade ≥ 2 GU toxicity. The current study has been designed to detect a 14% reduction in acute toxicity, from 29 to 15%. An overall sample size of 300 patients (150 per arm) will provide 83.7% power to detect an absolute risk reduction of 14% with a one-sided, two-sample Z-test at a 0.025 significance level. Assuming an accrual of 75–100 patients per year, patients will be accrued over a period of 3.5 years and follow-up will continue for 5 years after the last subject is enrolled. However, the primary endpoint would be available for analysis 90 days after the final patient enrolled completes treatment. A trial designed to detect a smaller absolute difference in toxicity would be prohibitively large as for the expected difference, a trial of 300 patients is already required. Also given the cost of acquiring and operating an MRI-guided LINAC, one could also argue that a smaller absolute difference in toxicity would question the clinical relevance of the modality.

The study will also evaluate differences in the rate of acute grade ≥ 2 GI toxicity as well as the 5-year cumulative incidences of late grade ≥ 2 GU and GI toxicity. Other secondary endpoints include 5-year BCRFS (which will be stratified by risk group) and the proportion of fractions delivered that required online adaptation. Given the assumed toxicity rates, the absolute differences in the secondary toxicity endpoints that could be identified with 80% power, one-sided alpha 2.5% with *n* = 150 patients per arm are provided in Table 3.

**Table 3:** Secondary endpoints power analysis

Secondary Endpoint	Assumed Rate in CT-Guided SBRT Arm	Absolute Difference at 80% Power (n = 150 per arm)
Acute grade ≥ 2 GI toxicity	16.0%	8.3%
Late grade ≥ 2 GU toxicity	13.3%	12.8%
Late grade ≥ 2 GI toxicity	2.0%	7.5%

### Data analysis

#### Analysis of primary Endpoin

The primary endpoint is the incidence of acute grade ≥ 2 GU toxicity on the CTCAE v 4.03 scale. Acute toxicity rates will be reported descriptively. Point estimates as well as the associated 95% confidence intervals (CIs) will be reported. Comparison between study arms will be performed via Chi-square test or fisher’s exact test.

#### Analysis of secondary endpoints

Secondary endpoints include the incidence of acute grade ≥ 2 GI toxicity on the CTCAE v 4.03 scale. Other secondary endpoints include 5-year cumulative incidences of late grade ≥ 2 GU and GI toxicity based on the CTCAE v 4.03 scale, which will be analyzed using a cumulative incidence framework. The analysis of both acute and late GI toxicity will be stratified for the use of hydrogel spacers or not, as these may reduce both acute and late GI toxicity.

Patient-reported outcomes on various QOL instruments will be other secondary endpoints of interest. Utilizing the EPIC-26 instrument, one can assess changes from baseline in domains such as urinary incontinence/ obstruction, bowel symptoms, sexual function, and hormone/vitality domains. Based on the magnitude of the changes, a determination can be made whether they represent minimally important differences [26]. As to the IPSS and SHIM QOL indices, both the numerical change from baseline, as well as the raw score at any given timepoint, will be collected and reported descriptively. A restricted maximum likelihood (REML)-based mixed models repeated measures (MMRM) approach will be used for analysis of QOL. The model will include time, fixed categorical effects of treatment, treatment-by-time interaction, and other fixed baseline covariates. In order to model the within-patient errors, an unstructured covariance structure will be employed. Kenward-Roger approximation will be used to estimate denominator degrees of freedom. The analysis of both acute and late changes in the bowel domain of the EPIC instrument will be stratified for the use of hydrogel spacers or not, as these may reduce both acute and late GI bowel symptoms. The impact of pelvic nodal irradiation on toxicities will be included as a pre-specified, albeit post-hoc analysis, as the decision is per investigator’s discretion pending achievable dosimetry, which may not be known until after randomization and simulation.

Kaplan-Meier statistics as well as descriptor statistics (such as mean, median, standard deviation, quartile range) will be ysed to estimate 5-year BCRFS. Figures showing the Kaplan-Meier estimates will also be presented. Death of any cause will be treated as a competing risk. Comparison of five-year BCRFS between study arms will be performed by log-rank test.

Point estimate and the corresponding 95% CIs will be calculated to determine the proportion of SBRT fractions for which online adaptive radiotherapy is required due to changes in OAR anatomy.

Imputation will not be used to handle missing data.

#### Early stopping guidelines

An interim safety analysis will be conducted after 2 years, or after 100 patients have been enrolled—whichever comes first— wherein we will also evaluate the actuarial rate of biochemical recurrence-free survival and examine the rate of acute or late grade ≥ 3 adverse GU and GI events. Interim reports will be prepared every six months until the results of the study are published. If the rate of grade ≥ 3 adverse GU or GI events is higher than 20%, accrual will be halted and the study subjected to careful review. If the rate is higher than 30%, the study will be terminated.

## Discussion

As of 2020, SBRT has become a guideline-concordant, standard of care option for patients with localized PCa. As with all forms of radiation for PCa, acute and late toxicities still occur. While severe adverse events are rare, moderate (i.e., grade ≥ 2) adverse events, particularly GU toxicities, occur in a fair number of patients. These are likely driven by radiation dose delivered to critical adjacent normal tissue. Aggressive urethral sparing in CT-guided SBRT has shown a considerable reduction in acute GU toxicity demonstrated by Zilli et al. [35]; however, certain key features of this practice such as routine use of a Foley catheter and endorectal balloon may limit the widespread adoption. The dose in that study (36.25Gy in 5 fractions) is also lower than in the present study. MRI-guided SBRT has multiple theoretical advantages in sparing critical adjacent normal tissue, including better visualization of the prostate itself, the ability for real-time tracking, and the ability to perform online adaptive radiotherapy to account for organ deformation. These will lead to a reduction in the planning margins required for prostate SBRT, in turn leading to reduced dose delivery to adjacent structures and, ideally, less toxicity. The MIRAGE trial is the first and only randomized trial to rigorously evaluate whether the theoretical advantages of MRI-guided radiotherapy for PCa translate into a meaningful clinical improvement. The trial is pragmatically designed to detect an improvement in acute GU toxicity. This endpoint was chosen due to the rapidity of the result and the frequency of the event – a trial based on changes in late GU toxicity would be impractically large and take years to report. Moreover, randomized trials evaluating technologies are well known to be incredibly challenging from an accrual standpoint due to well-documented patient and provider biases [36]. Indeed, randomized trials evaluating technological advances in radiation oncology are rare, and oftentimes novel technologies are adopted without rigorous prospective comparative studies. The MIRAGE trial will provide not only information on acute toxicities, but also well-curated and annotated information on late toxicities, oncologic efficacy, and patient-reported outcomes. We hope that the data from this trial, along with data from smaller, single-arm studies (NCT03664193, NCT03935308 and NCT02163317), will elucidate the benefit, if any, provided by the use of MRI-guidance for SBRT delivery.

## Supplementary Information

**Additional file 1: Table S1.** Organ-At-Risk Dose Constraints.

## Data Availability

The datasets generated and/or analyzed during the current study are not publicly available due to ongoing nature of the trial and possible compromise of individual privacy but are available from the corresponding author on reasonable request upon study conclusion. Results will be disseminated via publication or presentation.

## Abbreviations

SBRT: Stereotactic body radiotherapy
PCa: prostate cancer
MRI: Magnetic resonance imaging
CT: computed tomography
PET: positron emission tomography
IPSS: International Prostate Symptom Score
GU: genitourinary
GI: gastrointestinal
QOL: quality of life
YLD: years lived with disability
EBRT: external beam radiotherapy (EBRT)
NCCN: National Comprehensive Cancer Network
CTCAE: Common Terminology Criteria for Adverse Events
RTOG: Radiation Therapy Oncology Group
IMRT: intensity-modulated radiotherapy
LINAC: linear accelerator
CBCT: cone-beam CT
BCRFS: biochemical recurrence-free survival
EPIC-26: Expanded Prostate Cancer Index-26
SHIM: Sexual Health Inventory for Men
IIEF-5: International Index of Erectile Function-5
BCR: biochemical recurrence
MSKCC: Memorial Sloan Kettering Cancer Center
HIFU: high-intensity focused ultrasound
DOMStat: Department of Medicine Statistics Core
REDCap: Research Electronic Data Capture
GTV: gross tumor volume
CTV: clinical tumor volume
PTV: planning tumor volume
OAR: organ-at-risk
HT: Hormonal therapy.
CI: confidence interval
REML: restricted maximum likelihood
MMRM: mixed models repeated measures
