# Supplementary material for: Magnetic resonance imaging-guided stereotactic body radiotherapy for prostate cancer (mirage): a phase iii randomized trial
Source: BMC Cancer. 2021 May 11;21:538. doi: 10.1186/s12885-021-08281-x (PMC8114498; doi:10.1186/s12885-021-08281-x)
Supplement: Supplementary file 1 — Additional file 1: Table S1. Organ-At-Risk Dose Constraints. [file 12885_2021_8281_MOESM1_ESM.docx]

**Additional Table 1.** Organ-At-Risk Dose Constraints

| **Organ** | Volume | **Dose (cGy)** |
| --- | --- | --- |
| Rectum | 50% of total volume | 20 Gy |
|  | 50% of circumference on any given slice | 24 Gy |
|  | 20% of total volume | 32 Gy |
|  | 10% of total volume | 36 Gy |
|  | 5% of total volume | 40 Gy |
|  | Anterior rectal wall Maximum point dose* | 42 Gy (8.4 Gy per fraction) |
|  | Posterior rectal wall  Maximum point dose | 16 Gy (3.2 Gy per fraction) |
|  | Highest dose received by 2 cc | 38 Gy |
|  | 10 cc of rectum superior to the PTV | <25 Gy (5.0 Gy per fraction) |
| Anal Canal  (rectum inferior to any PTV) | Maximum point dose | 30 Gy (6.0 Gy per fraction) |
|  | Less than 5 cc | 20 Gy (4.0 Gy per fraction) |
| Small intestine | Maximum point dose | 25 Gy (5.0 Gy per fraction) |
|  | Less than 10 cc | 20 Gy (4.0 Gy per fraction) |
| Prostatic urethra | Maximum point dose within 1mm of target** | 42 Gy (8.4 Gy per fraction) |
| Bladder | Maximum point dose | No more than 105% of prescription dose |
|  | Less than 10 cc | 25 Gy (5.0 Gy per fraction) |
|  | Highest dose received by 2 cc | <39 Gy |
| Bladder Trigone | Maximum point dose | 42 Gy (8.4 Gy per fraction) |
| Femoral heads | Less than 10 cc cumulative (both sides) | 20 Gy (4.0 Gy per fraction) |
| Skin | Maximum point dose | 15 Gy (3.0 Gy per fraction) |
| Corpus cavernosum | Maximum dose to 5% of volume | 13 Gy (2.6 Gy per fraction) |
| Internal pudendal artery | Maximum dose to 5% of volume | 19.5 Gy (3.9 Gy per fraction) |
| Penile bulb | Maximum dose to 5% of volume | 24.8 Gy (4.96 Gy per fraction) |

*Maximum point dose here refers to the highest dose to 0.035 cc of the target.

**The urethra is only required to be considered as an avoidance structure for patients treated with MRI-guided SBRT. In cases where there is no biopsy-proven lesion within the transitional zone of the prostate and no MRI-identified gross lesion within 1 mm of the urethra, more aggressive urethral sparing (maximum point dose of 36 Gy within 1 mm of the urethra) is encouraged per physician discretion for patients receiving MRI-guided radiotherapy.
